# Analysis of Sinus Variability in a Group of Cameroonian Athletes

**DOI:** 10.1155/2024/1752677

**Published:** 2024-03-26

**Authors:** Deugoue F. Y. Djientcheu, M. Azabji-Kenfack, Poumeni M. Kameni, D. C. Bilanda, Membe U. Femoe, M. C. Ngoungoure, P. Kamtchouing, Djomeni P. D. Dzeufiet

**Affiliations:** ^1^Laboratory of Animal Physiology, Faculty of Sciences, University of Yaoundé I, Yaoundé, Cameroon; ^2^Laboratory of Physiology, Faculty of Medicine and Biomedical Sciences, University of Yaoundé I, Yaoundé, Cameroon

## Abstract

**Background:**

Heart rate variability (HRV) analysis is a useful method for assessing the heart's ability to adapt to endogenous and exogenous loads. Data from African population on HRV are scarce and even more so in sports populations. This study aimed to compare cardiac autonomic modulation response in Cameroonian athletes and sedentary. *Methodology*. We conducted a prospective and analytical study in sports teams in the city of Yaoundé, Cameroon. The participants in our study were divided in three groups; people who practiced little or no sporting activity (sedentary as group 1) or who were regularly physically active as part of a sports team (footballers or handballers as second and third groups). They had to be aged 18 or over and have given their informed consent. Heart rate (HR) was continuously recorded at rest for ten minutes and then transferred to a computer equipped with Kubios HRV Standard software for analysis. Means ± mean standard errors were compared using the one-way ANOVA test, followed by Tukey's post-test. The significance threshold was set at 0.05.

**Results:**

Of the 60 people selected to participate to our study, 75.0% were sportsmen (40.0% handball players and 35.0% footballers). The resting HR of sedentary people was higher (*p* < 0.001) than that of footballers and handball players. The SDNN, RMSSD, and pNN50 of sedentary people (16.22 ± 1.04; 9.97 ± 0.46; and 0.16 ± 0.06) were lower than those of footballers (30.13 ± 2.93; 20.61 ± 2.46; and 2.99 ± 0.63, with *p* < 0.001) and handball players (29.00 ± 1.86; 16.44 ± 1.16; and 2.15 ± 0.38, with *p* < 0.001 and *p* < 0.05 respectively). Absolute and relative very-low-frequency (VLF) power, absolute low and high-frequency (LF and HF) power, as well as total power (TP) were lower in sedentary people (3.66 ± 0.08 and 16.21 ± 0.64; 5.04 ± 0.15 and 2.50 ± 0.16 and 246.40 ± 18.04) compared to footballers (5.09 ± 0.24 and 26.87 ± 1.76; 5.85 ± 0.32 and 3.92 ± 0.22 and 836.10 ± 103.70, with *p* < 0.001, *p* < 0.01, and *p* < 0.001) and handball players (4.86 ± 0.16 and 30.82 ± 2.67; 6.03 ± 0.19 and 3.46 ± 0.16 and 927.30 ± 94.12, with *p* < 0.001, *p* < 0.05, *p* < 0.01, and *p* < 0.001). The LF/HF ratio was 12.1% and 20.1% lower in sedentary people (7.55 ± 0.58) compared with footballers (8.46 ± 0.50) and handball players (9.07 ± 0.60), respectively.

**Conclusion:**

Sportsmen showed greater parasympathetic and global modulation when compared to sedentary people.

## 1. Background

Healthy biological systems exhibit complex patterns of variability that can be described by mathematical chaos. The oscillations of a healthy heart are complex and constantly changing, giving the cardiovascular system the ability to rapidly adapt to sudden physical and psychological challenges for homeostasis [1]. The autonomic nervous system (ANS) plays a central role in the regulation of cardiovascular function. Nowadays, heart rate (HR) appears to be the most adequate indirect means to estimate metabolic demand during specific training [2, 3]. Heart rate variability (HRV) is produced by continuous changes in the sympathetic-parasympathetic balance which, in turn, causes the sinus rhythm to fluctuate around the mean HR [4]. Cardiovascular control mechanisms such as respiratory sinus arrhythmia, baroreflex or thermoregulation frequently make small adjustments to HR, leading to periodic HR fluctuations [4].

HRV refers to variations in interbeat intervals or instantaneous HR in different situations [3]. This change in the heart rate provides information about vagal and sympathetic modulation of cardiac functions [5]. It is a major noninvasive tool for assessing the influence of ANS activity at the cardiac level, reflecting the regulation of autonomic balance, blood pressure, gas exchange, gastrointestinal tract, heart, and vascular tone [3, 6]. At rest, HRV quantifies the intrinsic beat-to-beat changes in HR that occur through the vagus nerve to reflect the inhibitory actions of the parasympathetic nervous system (PNS) function on the sinoatrial node [1]. Given that the most fundamental component of HRV is due to the action of pacemaker cells in the sinoatrial node. This periodic action is continuously modulated by two competing branches of the ANS which is referred to as autonomic balance [7, 8].

Methods for quantifying HRV are classified into time, spectral or frequency, and geometric and nonlinear domains. Time domain indices quantify the level of HRV observed during monitoring periods that can range from about 2 minutes to 24 hours. This domain reflects the activity of the ANS and processes the R-R intervals over the recording period [9]. They are associated with the predominance of the PNS and as an overall indicator of the psychophysiological state of an athlete [10]. The frequency domain values calculate the absolute or relative amount of signal energy [1]. Its parameters can be modified as the ANS responds to changes in respiratory rate. Nonlinear measurements quantify the unpredictability and complexity of a series of interbeat intervals [1].

HRV can be influenced by many factors including physical activity [6, 11]. However, its use as a practical monitoring tool for allostatic load has been rare and could be a simple instrument for athletes. Higher HRV has been associated with lower cardiovascular risk, better fitness and responsiveness to aerobic training, higher levels of physical activity, and reduced work-related stress [6]. Nevertheless, low HRV values indicate an inefficient ANS, leading to maladaptive responses to stress and perceived threats [3].

HRV analysis has been established as a useful method to assess the capacity of the heart to adapt to endogenous and exogenous loads [12]. It can be used for the individual assessment of responses to training loads. Indeed, in recent years, HRV has been used to evaluate different aspects related to training such as recovery and overtraining, exercise intensity and duration, training load, or psychophysiological profiles [3, 13]. However, very few studies have been done in sub-Saharan Africa regarding HRV and are particularly interested in subjects suffering from various pathologies. In this way, it has been shown that parasympathetic activity is impaired in sub-Saharan patients with hyperthyroidism and that cardiac autonomic modulation is weak in patients with poorly controlled type 2 diabetes [14, 15]. This study aimed to compare cardiac autonomic modulation response in Cameroonian athletes (footballers and handball players) and sedentary.

## 2. Materials and Methods

### 2.1. Participants and Study Design

We conducted a prospective and analytical study on several sports teams in the city of Yaoundé (Cameroon). The participants were active sportsmen, aged at least 18 years, and have given their consent. All those who gave their consent were included in the study. These participants were required to have been regularly physically active (football or handball) for at least one year as part of a team (Sportsmen group) or to have done little or no physical activity at all (Sedentary group). In addition, conditions such as symptoms of heart and/or lung disease, chronic illnesses such as hypertension, diabetes, or kidney disease were considered exclusion criteria. Each participant was required to have complete data and no participant had taken any medications and a history or illicit drug use and all them were nonsmokers.

Age and anthropometric parameters (weight and height) were recorded and the Body Mass Index (BMI) was calculated. This study received the ethical clearance N°1587 CE/CRERSHC/2020 from the Regional Research Ethics Committee for Human Health of the Centre and was conducted with strict respect for the integrity of the participants, as well as respecting the pandemic barrier measures against COVID-19. Participants had the right to take out from the study at any time.

### 2.2. HRV Analysis

The recording took place at least 24 hours after any significant physical activity as well as the consumption of alcohol or tobacco. The participants, without any electronic equipment, were asked to maintain the position indicated and were not allowed to interact in any way during the recording and the ten minutes of prerecording rest. HR was continuously recorded at rest in a seated position for ten minutes in a quiet environment at room temperature [16, 17] using an HR monitor (POLAR H10®, *Polar Electro Inc., Singapore*).

The data collected were stored in a smartphone equipped with the *HR* and *HRV Logger*® application. At the end of the recording, they were transferred to the *Kubios HRV Standard*® software version 3.3.3 installed in a desktop computer for analysis. The fast Fourier transformation method was used to perform a power spectral analysis. The HR monitor data were converted and transferred to *Microsoft Excel 2013*® software where the HR values were converted to RR intervals (in milliseconds).

The resulting tachogram was divided into two five-minute phases from which we extracted the second phase and transferred it into the *Kubios HRV Standard*® software for analysis of HRV parameters. These parameters were represented by the time domains (the standard deviation of the NN interval (SDNN), the square root of the mean of the sum of the squares of differences between adjacent NN intervals (RMSSD), and the proportion derived by dividing the number of interval differences of successive NN intervals greater than 50 ms by the total number of NN intervals (pNN50)), frequency (VLF, HF, LF, LF/HF ratio, and TP) and nonlinear (SD_1_, SD_2_, and SD_2_/SD_1_ ratio). These HRV parameters were then exported and stored in Microsoft Excel to form our database.

### 2.3. Statistical Analysis

The data obtained were analyzed by *SPSS* version 23.0 (IBM SPSS® Inc, Chicago, Illinois, USA) and GraphPad Prism version 8.0 (GraphPad Software, San Diego, California, USA). Means ± mean standard errors (MSEs) were compared using the one-way ANOVA test, followed by Tukey's multiple comparison post-test. A one-way ANCOVA was performed to compare the impact of sports practice on certain HRV parameters, while controlling for age and baseline HR, using the Levene test or its nonparametric equivalent, followed by the Bonferroni post-test. The significance threshold was set at *p* < 0.05.

## 3. Results and Discussion

### 3.1. Results

#### 3.1.1. Study Population

**Figure 1:**
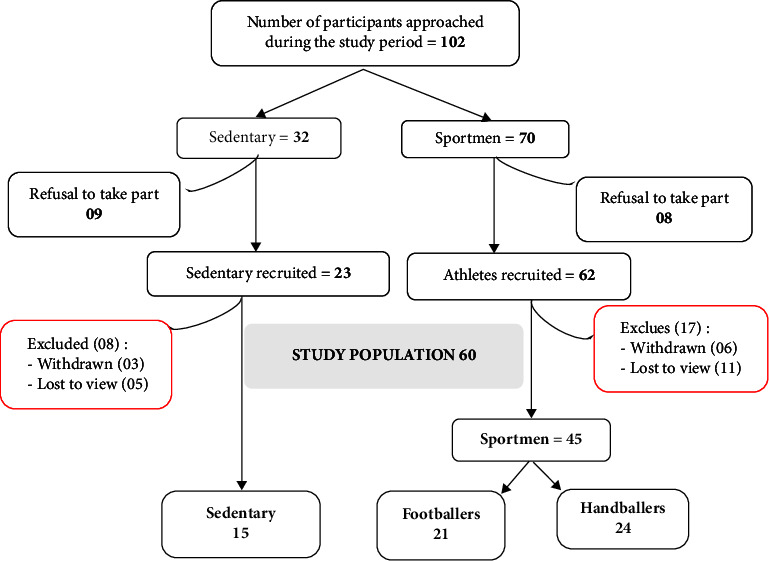
Study flowchart.

For this study, we approached 102 potential participants in the community and sports structures. Of the 60 people selected to participate in our study, 75.0% (45/60) were sportsmen (40.0% handball players (24/60) and 35.0% football players (21/60)), opposite to 25.0% (15/60) sedentary people (Figure 1).

#### 3.1.2. Clinical and Sporting Characteristics of the Study Population

*(1) Clinical*. Handball players were significantly older than footballers (*p* < 0.05). The resting HR of sedentary people was significantly higher (*p* < 0.001) than that of sportsmen (*p* < 0.001) (Table 1).

**Figure 2:**
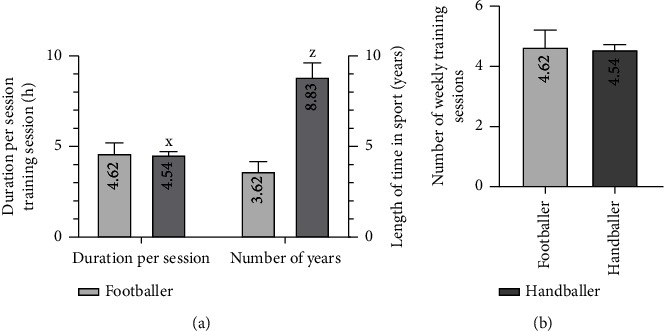
Distribution of sportsmen according to their sports practices: (a) duration per session and number of years of sports practice and (b) number of weekly training sessions. Each bar represents the mean ± MSE. ^x^*p* < 0.05 and ^z^*p* < 0.001 denote significant difference from footballers.

*(2) Sports Practice*. Concerning the sports practice of the participants in our study, handball players had significantly more years of sports practice, with significantly higher durations per training session than football players (*p* < 0.001 and *p* < 0.05) (Figure 2(a)). There was no significant difference between the two groups of athletes in the number of weekly training sessions (Figure 2(b)).

#### 3.1.3. Characteristics of Sinus Variability

**Figure 3:**
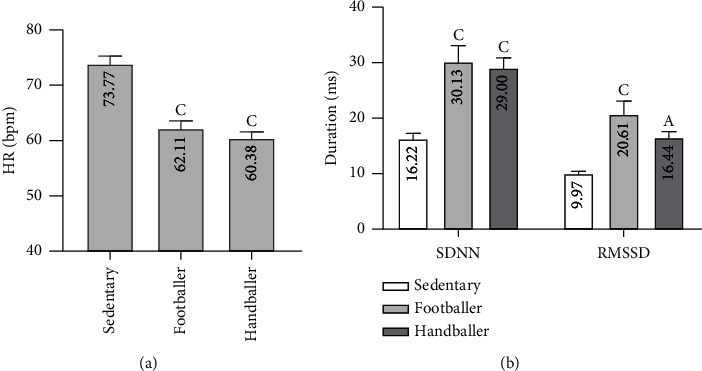
Distribution of the population according to time domain analysis: (a) resting heart rate and (b) SDNN and RMSSD. Each bar represents the mean ± MSE. ^A^*p* < 0.05 and ^C^*p* < 0.001 denote significant difference from sedentary people. HR: Heart rate; RMSSD: root mean square of the successive differences; SDNN: the standard deviation of the interbeat interval of normal sinus beats.

*(1) Time Domain Analysis*. The resting HR of sedentary people was significantly higher (*p* < 0.001) than that of footballers and handballers (Figure 3(a)). The SDNN, RMSSD, and pNN_50_ of sedentary people were significantly lower (*p* < 0.001) than those of footballers and handballers (*p* < 0.001, *p* < 0.05, and *p* < 0.05, respectively). But there was no significant difference between the sports groups (Figure 3(b) and Table 2).

*(2) Frequency Analysis*.

- Low and very low frequencies.
- The absolute and relative power of VLF were significantly lower in sedentary people than in footballers (*p* < 0.001 and *p* < 0.01, respectively) and handball players (*p* < 0.001). The absolute power (ms^2^) of the LF was significantly greater in footballers (*p* < 0.01) and handballers (*p* < 0.001) compared to sedentary people, while the relative power of sedentary people was significantly higher than that of footballers (*p* < 0.01) (Table 3).
- High frequency and normalized power.
- The absolute power (log) of HF in sedentary people was significantly lower than that of footballers (*p* < 0.001) and handball players (*p* < 0.01) (Table 3).
- The TP of sedentary people was significantly lower than that of footballers and handball players (*p* < 0.001). The LF/HF ratio was 12.1% and 20.1% lower in sedentary people compared to footballers and handball players, respectively (Table 3).
- Nonlinear analysis.
- Although the SD_1_ and SD_2_ indices were lower in the sedentary population (*p* < 0.001) compared to footballers and handball players, we did not find any significant difference between the different groups concerning the SD_2_/SD_1_ ratio (Table 3).

#### 3.1.4. Covariance Analysis

After controlling for age and baseline HR, there was a significant difference in SDNN (*F* (2, 55) = 5.978, *p* < 0.01), RMSSD (*F* (2, 55) = 3.606, *p* < 0.05), and TP (*F* (2, 55) = 7.158, *p* < 0.01) concerning the level of sporting activity. Post hoc showed less significant and even nonsignificant differences in SDNN, RMSSD, and TP between sedentary people and footballers (*p*=0.005, *p*=0.032, and *p*=0.008, respectively, in contrast to *p*=0.0006, *p*=0.0003, and *p*=0.0002 initially) and between sedentary and handball players (*p*=0.012, *p*=0.420, and *p*=0.002, respectively, in contrast to *p*=0.0002, *p*=0.042, and *p* < 0.0001 initially) (Table 4).

In addition, the adjusted means clearly show that the sedentary group lost in SDNN (*M* = 15.64 vs. 16.22) and gained in RMSSD (*M* = 9.97 vs. 11.14) and TP (*M* = 255.62 vs. 246.41), while the opposite trends were noted for the other groups. Lastly, the effect size for sports activity was low for these three parameters. The partial Eta squared value showed that 12%, 18%, and 21% of the variance in RMSSD SDNN, and TP, respectively, was explained by sports activity (Table 4).

### 3.2. Discussion

This study aimed to compare cardiac autonomic modulation response in Cameroonian athletes and sedentary. Our results show that the resting HR of sedentary people was higher (*p* < 0.001) than that of footballers and handball players. The SDNN, RMSSD, and pNN_50_ of sedentary people were lower (*p* < 0.001) than those of footballers and handball players (*p* < 0.001, *p* < 0.05, and *p* < 0.05, respectively). Absolute and relative very-low-frequency (VLF) power, absolute low and high-frequency (LF and HF) power, as well as total power (TP) were lower in sedentary people compared to footballers (*p* < 0.001, *p* < 0.01, and *p* < 0.001) and handball players (*p* < 0.01 and *p* < 0.001). The LF/HF ratio was lower in sedentary people compared with footballers and handball players. Furthermore, the effect size for sports activity was low for RMSSD, SDNN, and TP.

HR during intensive training has shown significant reductions in athletes [18]. This is the case in this study where the resting HR of sedentary people was higher than that of athletes. This result could be explained by hyperactivation of the PNS via central, cardiac, and/or peripheral mechanisms as suggested by some authors [19, 20]. This decrease in resting HR in athletes could also be due to training, which could induce an increase in plasma volume, allowing for a greater stretch of the heart and an increase in stroke volume, as well as a greater sensitivity of the PNS, thus improving the physical condition of the subject undergoing this training [18, 21]. Indeed, optimal physical conditioning, coupled with adequate recovery, creates a healthy “imbalance” between the activities of the sympathetic and parasympathetic nervous systems in favour of greater vagal dominance. This is probably due to an increase in parasympathetic activity and eventually a decrease in sympathetic activity [22]. In addition, training may decrease the intrinsic rate of sinus node firing [23], which explains the observed decrease in HR in the athletes in our study.

The lower SDNN and RMSSD values of the sedentary compared to the athletes in this study could be explained by the difference in fitness between the athletes in our study and the nonathletes. In fact, Buchheit et al. [24] showed that there is a relationship between HRV exercise and performance improvement, with increased HRV after a boot camp. Furthermore, high HRV values indicate greater parasympathetic activation than sympathetic activation in an athlete and, therefore, a better ability to respond to subsequent demands [3, 25]. We found no significant difference between the different groups of athletes concerning SDNN, RMSSD, and pNN_50_. This minimal variation in the cardiac autonomic nervous system activity suggests that the training program of these athletes incorporated comparable intensity, load, rest, and recovery [26, 27]. This similarity in training levels may have mitigated any changes in ANS activity between groups of athletes [27].

The low values of the temporal analysis parameters (SDNN and RMSSD) obtained in our study compared to those documented in the literature could be a consequence of low recovery time and/or reflect a state of fatigue [1, 13]. Participants had less than 24 hours of recovery from their previous training session before participation in this study. Moreover, HRV may be reduced, due to sympathetic dominance, in the days following intense exercise [18]. This low variance in R-R intervals is often associated with a decrease in baroreceptor reflex sensitivity and suggests a possible alteration in cardiac vagal tone regulation [28]. However, these changes in the amplitude of temporal cues are transient and are quickly corrected after good recovery [29].

Furthermore, it had been reported that in endurance sports such as those practiced by the participants in our study, there is a sensitivity of HRV to training and competition that can be improved when data are averaged over a week compared to using single data points as in our study. This is because of the high daily variation in these indices [30]. However, it is not obvious to undertake such measurements with the high number of athletes involved in team sports. Nonetheless, future research should consider how such approaches might improve the suitability of these measures for use in wider team sports and other populations.

Regardless of the modality chosen, VLF had higher power in athletes compared to sedentary people. These results are consistent with the work of other authors who have shown that VLF power and overall HR variability are increased by physical activity. Such fluctuations in VLF of R-R intervals that are generated by physical activity have been attributed to a variety of factors including the renin-angiotensin system, thermoregulation, chaotic regulation of the cardiac period, and endothelial influences on the heart [1, 31]. In the same way, the frequency and absolute power of LF were increased in athletes compared to sedentary people. Similar results were obtained by other authors [23, 26] who then suggested that intensive training would be able to induce a reduction in parasympathetic modulation and an increase in cardiac sympathetic activity during rest. However, this interpretation must be qualified in the light of the work of Billman [32] who reported that the LF component of HRV more often reflects a complex combination of sympathetic and parasympathetic factors as well as other unidentified factors, with definite implications for the interpretation of the LF/HF ratio [32].

The HF spectrum was greater in athletes than in sedentary people. Such sport-related effects are thought to be the result of adaptations of various systems, including the central nervous system, with decreased sympathetic tone [23]. The low level of HF in sedentary people would also be related to their higher blood pressure and shorter intervals between their R waves [33]. Furthermore, the differences observed in the HF spectrum between footballers and handball players could be related to variations in the respiratory rate and volume which can modify to greater or lesser extent HRV indices such as HF power, but also pNN_50_ or RMSSD, without anyway affecting the vagal tone [1].

The LF/HF ratio is used as an indicator of the balance between the different systems involved (sympathetic and parasympathetic). In this study, this ratio was higher in athletes than in sedentary people. The hypothesis supporting the LF/HF ratio is that LF power is generated by the sympathetic nervous system (SNS) while HF power results from the activity of the PNS. Consequently, a high LF/HF ratio as observed in the athletes in our study indicates sympathetic dominance, which also occurs when we engage in parasympathetic fight or flight or withdrawal behaviors [1]. In our study, an increase in the LF/HF ratio was associated with a greater increase in LF indices of HRV. Buchheit et al. [26] found similar results and showed that this was associated with a decrease in Ln HFRR and Ln BRS. These authors also suggested that these findings would result from a decrease in parasympathetic modulation and an increase in sympathetic activity respectively. Furthermore, it has been reported that half of the variability in the LF frequency band is due to the SNP. Also, the interactions between PNS and SNS are complex, nonlinear, and often nonreciprocal and finally, the confounding by respiratory mechanics and resting HR creates uncertainty about the contributions of PNS and SNS to the LF/HF ratio during the measurement period [1]. All this puts the reliability of the LF/HF ratio measurement as an indicator of sympathovagale balance into perspective.

The significant increase in TP of athlete's groups compared with sedentary controls, which persisted even after adjustment with age and resting HR, associated with the increase in HF observed here, would be linked to an increase in HRV, particularly in the HF spectrum, as well as the reduction in resting HR. This may result in cardiac autonomic adjustments as well as greater parasympathetic modulation as a result of sport [23].

The elevation of SD_1_ and SD_2_ values in athletes compared to sedentary participants would reflect short- and long-term improvements in HRV after different early-season training regimes in athletes [24]. Although we did not find previous reports showing the effects of training on resting cardiac autonomic control in Cameroonian team sports athletes and there is very little evidence in the international literature, HRV has been shown to improve with training in team sports athletes [24]. Moreover, Thorpe et al. [18] found that in Australian Football League players training before the season, SD_1_, a parameter of HRV related to vagal tone [34], was correlated with daily training load. These unexpected changes in parasympathetic activity may have been partially mediated by thermoregulatory mechanisms associated with alterations in plasma volume [18].

In general, the observed increase in power in the different individual frequency bands and TP, as well as relevant values in the time and nonlinear domains would reflect the good physical conditioning of the athletes in our study [1]. Nevertheless, the explanation for the controversial results observed between the present study and the literature data would probably come from the fact that the precise physiological mechanisms that influence HRV are not fully elucidated [9]. As a result, different study protocols could influence or alter one parameter or another, without having a proven physiological basis.

## 4. Conclusion

The athletes in this study showed higher values of HRV parameters, in particular an increase in vagal tone compared with sedentary people.

### 4.1. Limitations of the Work

The main limitations of this work were the lack of observance of the participants to the study condition including strict rest and nonverbal interaction with others which did not allow us to have the desired level of precision. We were also powerless to control the temperature and humidity conditions in the experimental room. The participants were not familiar with the collection environment before recording the data.

## Figures and Tables

**Table 1:** Distribution of the study population according to their clinical data.

Variables	Sedentary (*n* = 15)	Sportsmen	
		Footballers (*n* = 21)	Handballers (*n* = 24)
Age (years)	26.27 ± 1.25	23.76 ± 0.82	29.46 ± 1.05^z^
BMI (kg/m^2^)	26.38 ± 0.94	24.36 ± 0.62	25.20 ± 0.75
DBP (mmHg)	69.27 ± 1.93	71.29 ± 1.49	76.25 ± 2.04
SBP (mmHg)	124.90 ± 3.08	117.00 ± 1.46	122.80 ± 2.58
MBP (mmHg)	87.80 ± 1.70	86.52 ± 1.25	91.76 ± 2.06
Rest HR (bpm)	73.47 ± 2.22	59.19 ± 1.20^c^	59.83 ± 1.40^c^

Each value represents the mean ± MSE, ^c^*p* < 0.001denotes significant difference from sedentary people, and ^z^*p* < 0.001 denotes significant difference from footballers. HR: heart rate; BMI: body mass index; DBP: diastolic blood pressure; MBP: mean blood pressure; SBP: systolic blood pressure.

**Table 2:** Distribution of the population according to the time analysis.

Parameters	Sedentary (*n* = 15)	Sportsmen	
		Footballers (*n* = 21)	Handballers (*n* = 24)
RR (ms)	840.00 ± 8.83	979.90 ± 24.96^c^	994.50 ± 24.91^c^
Min HR (bpm)	68.30 ± 1.32	54.95 ± 1.48^c^	54.73 ± 1.28^c^
Max HR (bpm)	77.77 ± 1.10	77.17 ± 1.41	74.26 ± 1.91
NN_50_ (beats)	0.87 ± 0.34	8.19 ± 1.86^b^	5.33 ± 1.01
pNN_50_ (%)	0.16 ± 0.06	2.99 ± 0.63^c^	2.15 ± 0.38^a^

Each value represents the mean ± MSE, ^a^*p* < 0.05. ^b^*p* < 0.01 and ^c^*p* < 0.001 denote significant difference from sedentary. Min and Max HR: minimum and maximum registered heart rate at rest; NN_50_: the number of pairs of successive NN (R-R) intervals that differ by more than 50 ms; pNN50: the proportion of NN50 divided by the total number of NN (R-R) intervals; RR: duration between two consecutive R waves.

**Table 3:** Repartition of the study population according to the parameters of the frequency analysis.

Variables	Sedentary (*n* = 15)	Sportsmen	
		Footballers (*n* = 21)	Handballers (*n* = 24)
VLF			
Absolute power (ms^2^)	45.27 ± 2.90	281.90 ± 69.27^c^	200.70 ± 29.53
Absolute power (log)	3.66 ± 0.08	5.09 ± 0.24^c^	4.86 ± 0.16^c^
Relative power (%)	16.21 ± 0.64	26.87 ± 1.76^b^	30.82 ± 2.67^c^
LF			
Absolute power (ms^2^)	170.80 ± 16.31	603.80 ± 100.80^b^	658.30 ± 83.09^c^
Absolute power (log)	5.04 ± 0.15	5.85 ± 0.32	6.03 ± 0.19^a^
Relative power (%)	75.35 ± 0.86	64.64 ± 1.51^b^	69.26 ± 2.63
Normalized powers (n.u.)	87.23 ± 1.47	88.30 ± 1.09	89.18 ± 1.24
HF			
Absolute power (ms^2^)	22.95 ± 1.33	78.52 ± 13.16^c^	42.76 ± 5.15^y^
Absolute power (log)	2.50 ± 0.16	3.92 ± 0.22^c^	3.46 ± 0.16^b^
Relative power (%)	8.39 ± 0.38	8.61 ± 0.51	6.96 ± 0.42
Normalized powers (n.u.)	12.79 ± 1.18	11.14 ± 0.62	10.90 ± 0.76
Normalized powers			
Total power (ms^2^)	246.40 ± 18.04	836.10 ± 103.70^c^	927.30 ± 94.12^c^
Ratio LF/HF	7.55 ± 0.58	8.46 ± 0.50	9.07 ± 0.60
Nonlinear analysis			
SD_1_ (ms)	5.89 ± 0.34	13.56 ± 1.57^c^	11.44 ± 0.84^b^
SD_2_ (ms)	20.14 ± 0.83	36.48 ± 2.81^c^	36.32 ± 2.40^c^
Ratio SD_2_/SD_1_	3.50 ± 0.14	3.37 ± 0.46	3.31 ± 0.19

Each value represents the mean ± MSE, ^a^*p* < 0.05. ^b^*p* < 0.01 and ^c^*p* < 0.001 denote significant difference from sedentary people, and ^y^*p* < 0.01 denotes significant difference from footballers. HF: high frequency; LF: low frequency. SD_1_ and SD_2_: standard deviations of the scattergram; VLF: very low frequency.

**Table 4:** Repartition of the study population according to the ancova analysis.

Variables	Adjusted mean			*F*	df	*η* ^2^ value	*p* value
	Sedentary	Footballers	Handballers				
SDNN	15.64 ± 3.36	30.49 ± 2.47^b^	29.05 ± 2.36^a^	5.978	2–55	0.179	0.004
RMSSD	11.14 ± 2.54	20.04 ± 1.87^a^	16.21 ± 1.78	3.606	2–55	0.116	0.034
TP	255.62 ± 136.50	828.36 ± 100.37^b^	928.30 ± 95.70^b^	7.158	2–55	0.207	0.002

Each value represents the mean ± MSE, ^a^*p* < 0.05. ^b^*p* < 0.01 and ^c^*p* < 0.001 denote significant difference from sedentary people. df: degree of freedom; *η*^2^: partial Eta squared value; RMSSD: root mean square of the successive differences; SDNN: the standard deviation of the interbeat interval of normal sinus beats; TP: total power.

## Data Availability

The data used to support the findings of this study are included within the article. Raw data are available on request from the corresponding author.
